# Testicular needle aspiration: Is it a safe method for breeding soundness evaluation in bulls?

**DOI:** 10.21451/1984-3143-AR2019-0007

**Published:** 2019-11-18

**Authors:** André Maciel Crespilho, Cristiano Silva Ferreira, Yolanda Henrichs Garcia Bandeira Bastos, Priscilla Nascimento Guasti, José Carlos Nascimento, Kátia de Oliveira Pimenta Guimarães, Rafael Garabet Agopian, Gustavo Mendes Gomes, Kleber da Cunha Peixoto

**Affiliations:** 1 Universidade Santo Amaro, Programa de Pós-graduação em Medicina Veterinária e Bem-Estar Animal, São Paulo, SP, Brasil; 2 Universidade de Vassouras, Vassouras, RJ, Brasil; 3 Faculdade de Ensino Superior e Formação Integral, Curso de Medicina Veterinária, Garça, SP, Brasil

**Keywords:** bovine, seminiferous tubule, degeneration, testicular puncture

## Abstract

The aim of this study was to evaluate the impact of successive bovine testicular punctures using different needle sizes. Fifteen bulls were submitted to testicular needle aspiration (TNA) in the left and right testis using 18-gauge (40×12mm) or 22-gauge (25×7mm) needles, respectively, once every 30 days. Animals were randomly divided into three groups, which were submitted to bilateral orchiectomy two days after the last puncture. Group 1 (G1): only one puncture (n=5); Group 2 (G2): three consecutive punctures in a period of three months (n=5); Group 3 (G3): six consecutive punctures in a period of 6 months (n=5). Fragments from the medial portion of the testicular parenchyma were excised and fixed in Bouin’s fluid for histological analysis. No differences were observed in the percentage of seminiferous tubules degeneration between G1, G2 and G3 (P>0.05). Higher amounts of erythrocyte were found in G1 and G2 groups compared to G3, in the intra- and intertubular tissue (P<0.05). There was no interaction between the needle gauge and the occurrence of testicular damage in animals submitted to one (G1) or three (G2) punctures. However, a higher percentage of tubular degeneration was associated to 18-gauge compared to 22-gauge fine needles in G3. In conclusion, multiple testicular needle aspiration can be safely conducted using fine needles. Large needles are recommended only for a single TNA, since multiple punctures may result in increased tubular degeneration and compromise testicular architecture and functionality.

## Introduction

The biopsy procedure using needle aspiration began in studies with human medicine in the 1930s and is currently used to evaluate lesions of the mammary gland (Frankel et al., 2011; Freitas et al., 2006; Kemp et al., 2001) and to monitor spermatogenesis in assisted reproduction programs (Gilman et al., 2018; Jha and Sayami, 2009).

Testicular needle aspiration (TNA) is considered a safe, minimally invasive, quick and inexpensive technique that allows a significant number of punctures, obtaining a more complete testicular evaluation and providing objective information about spermatogenesis (Gouletsou et al., 2012). It is also an auxiliary method for the diagnosis of male infertility, being considered as a technique of choice in relation to biopsy due to the lower risk of testicular lesion that may compromise the reproductive life of an animal (Paz et al., 2003).

Although TNA is considered a low-cost and fast-performing technique, there is no consensus on the best needle diameter or risk of lesion of the testicular parenchyma undergoing serial punctures when using this technique in cattle. In dogs, successive TNA has no effect on spermatogenesis and plasma testosterone concentration, indicating the safety of punctures for investigations of reproductive diseases (Souza et al., 2004).

In humans, previous studies using TNA indicated that needle diameter exerts a significant influence on the recovery of biological material. Carpi et al. (2005) observed that the number of seminiferous tubules recovered in TNA with large needle was similar to the amount that could be obtained through open surgery in men with nonobstructive azoospermia. Moreover, the fine (22 gauge) and large-needle (18 gauge) testicular puncture did not produce clinical or subclinical complications; however, needle diameter influenced the quality of spermatogenic cell identification, in which fine needle aspiration was more adequate in identification spermatids and elongated spermatozoa (Carpi et al., 2005; Carpi et al., 2006).

Although studies in human andrology demonstrate the safety of puncture techniques to obtain testicular samples, the lesions that repeated punctures can cause to the testes of animal breeders remain unknown (Gouletsou et al., 2010). Therefore, the aim of this study was to evaluate the possible side effects related to the use of successive punctures performed with hypodermic needles of different gauges in the bovine testicular architecture.

## Methods

### Local and animals

This study was conducted at Santa Cecilia Farm, located in Vassouras, RJ, Brazil (22º 24 '9” South latitude and 43º 39' 8” West longitude, average altitude 434 m). All animal procedures were approved by the Institutional Ethics Committee of Vassouras University (USS, Vassouras, RJ, Brazil), Protocol Number 005/2014, according to Brazilian Council for the Control of Animal Experimentation (CONCEA). Fifteen crossbreed Girolando bulls aged 24 to 30 months were kept in pasture with free access to water and mineral salt. Before the experiment, animals were submitted to general clinical examination and specific evaluation of the reproductive system.

### Testicular needle aspiration

Animals were submitted to monthly TNA, according to specific treatment, in the left and right testes using 18-gauge (40×12mm; large needle aspiration) or 22-gauge (25×7mm; fine needle aspiration) hypodermic needles (Descarpack™, São Paulo, Brazil), respectively.

The hypodermic needles were coupled to 20-mL syringes (Descarpack™, São Paulo, Brazil) and, after previous topical antisepsis with povidone-iodine solution, the punctures were performed perpendicular to the longitudinal axis of each testis, in the medial region between the cranial and caudal extremities. After insertion of the needle into the testicular parenchyma, complete traction of the plunger was performed to create the necessary vacuum for the aspiration of spermatogenic cells. In each TNA, the hypodermic needle was moved 3 times outward and into each gonad (without removing the needle from the testis, respecting the axis of insertion) to promote the necessary cellular dislocation, according to the methodology described by Leme and Papa (2010).

### Orchiectomy

Animals were randomly divided into three groups; which were submitted to bilateral orchiectomy two days after the first puncture (G1, n=5, 1 puncture); two days after the three consecutive monthly punctures (G2, n=5, 3 punctures) and two days after six consecutive monthly punctures (G3, n=5, 6 punctures) on each testis.

Prior to castration, the animals were subjected to 24 hours of solid fasting and sedated with xylazine hydrochloride (0.05 mg/kg IV, Xilazin™, Syntec, Brazil). Local anesthetic blockage was performed with 10 mL of 1% lidocaine (Anestt™, Syntec, Brazil), applied to each spermatic cord.

The animals were kept standing in a cattle squeeze chute with abdominal belts. Antisepsis of the scrotum and inguinal region were performed with topical application of povidone-iodine solution. Orchiectomy was performed by open technique with longitudinal incision in the scrotum and the surgical hemostasis with 3-0 chromic catgut ligature (Brasuture™, São Sebastião da Grama, Brazil) in each spermatic cord.

Postoperative care was similar in all experimental groups, including the topical application of repellent (Lepecid Spray™, Ouro Fino Animal Health, Brazil) and single dose administration of a combination of penicillins, dihydrostreptomycin sulfate and piroxicam (Pencivet Plus™, MSD Animal Health, São Paulo, Brazil).

### Histological evaluations

After castration, each testis was hygienized with sterile 0.9% sodium chloride solution. Then, the testes were sectioned longitudinally for macroscopic analysis and fragments were collected for histological preparation. The tissue fragments were excised from the middle portion of each testis to coincide with the path of the needles during the puncture and aspiration.

For histological processing, adapted from Moura and Erickson (2001), tissue samples were fixed in Bouin’s solution for 24 hours and then immersed in 70% ethanol for 48 hours. The fragments were embedded in paraffin and sections of 4-micron thickness were stained with hematoxylin and eosin.

The seminiferous tubules of each sample were evaluated for the degree of tissue injury defined by the presence of erythrocytes within (intratubular hemorrhage) or outside (parenchymal or extra tubular hemorrhage) of the testicular lumen, tubular enlargement and degeneration, according to methodology adapted from Gouletsou et al. (2012). All analyzes were performed using light microscopy under 100× magnification,

### Statistical analysis

The results are expressed as mean ± SD. All data were analyzed using SAS statistical package (SAS Institute Inc., Cary, USA). The normality of the residues was evaluated by Shapiro-Wilk test (Proc-Univariate) and the homogeneity of the variances was compared by Chi-Square test (Proc GLM Spec command).

The effect of the groups (1, 3 or 6 testicular punctures), treatments (needle size 40×12 mm or 25×7 mm) and their possible interactions in the occurrence of testicular histological changes considered as dependent variables, were compared using the general linear model of variance (Proc GLM), using means adjusted through the “LS-means” command of the SAS. Differences were considered significant when P<0.05.

## Results

All animals were clinically evaluated after TNA and showed no signs of pain or inappetence. During the puncture procedure, no discomfort or visible inflammatory process was observed, independently of the experimental group. As G1 animals were castrated two days after the first puncture, it was not possible to perform a continuous evaluation of the side effects of the punctured testes. However, no changes in scrotal circumference were observed after the multiple TNA in G2 (P=0.8525) or G3 (P=0.5647).

**Figure 1:**
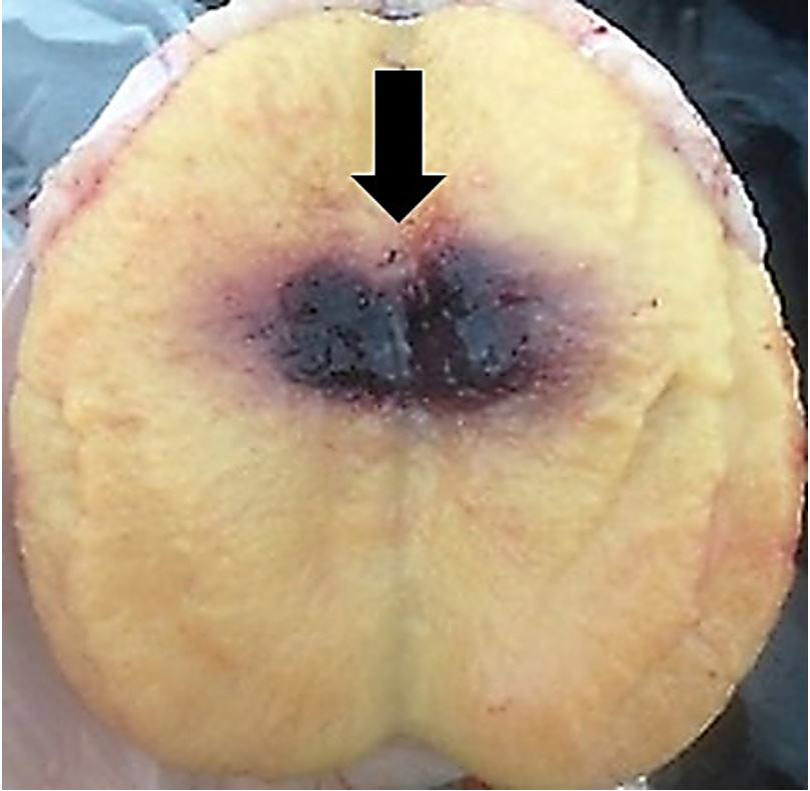
Longitudinal section of the bovine testicular parenchyma demonstrating focal hemorrhagic area (arrow) two days after Testicular Needle Aspiration (TNA). Testicle submitted to three TNA every 30 days (G2) using 40x12 mm (18G) needle.

All animals presented complete healing of the surgical wound within 7 days after orchiectomy, without intercurrences, except for two G1 animals, in which a discrete hematoma was observed in the vaginal cavity. However, no adhesions were detected between the visceral and parietal vaginal tunic in any of the evaluated animals. Interestingly, intratesticular haemorrhage on the medial surface was observed in all orchiectomized testes two days after TNA (Figure 1).

**Figure 2:**
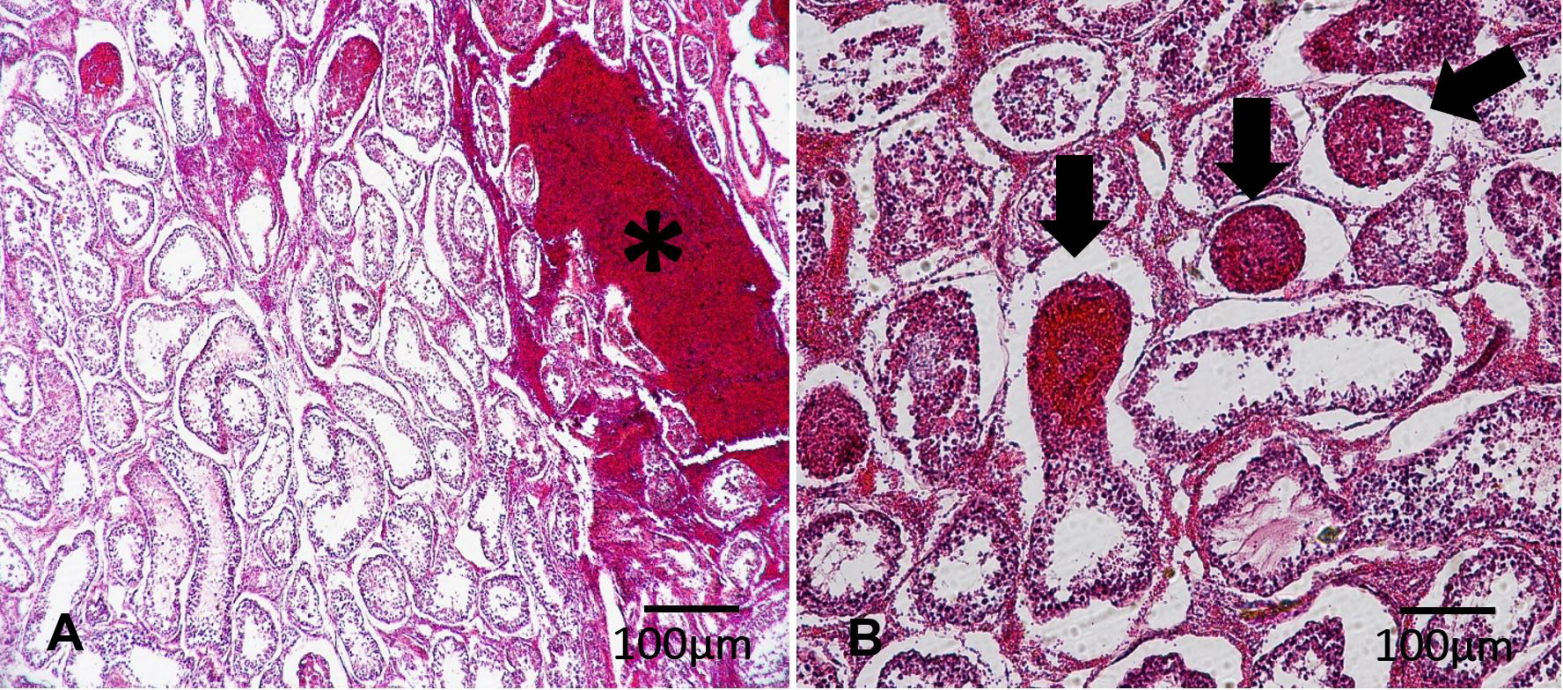
Photomicrographies of bovine testicular parenchyma after testicular needle aspiration illustrating the presence of intertubular hemorrhage (A, asterisk) and intratubular hemorrhage (B, arrows). Haematoxylin and eosin stain.

For histological evaluations, differences were observed in the percentage of intratubular (3.67, 2.60, 0.55; P=0.0020) and intertubular hemorrhage (22.33, 21.20, 3.33; P=0.0368), respectively for G1, G2 and G3, regardless of the needle gauge used (Figure 2). However, no difference in intratubular hemorrhage (2.35, 2.69; P=0.8981) and intertubular hemorrhage (17.35, 19.61; P=0.6330) was observed when TNA was performed with large (18-gauge) or fine (22-gauge) needle, respectively. The intra and intertubular hemorrhage percentage in groups G1, G2 and G3, according to the needle size, are described in Table 1.

**Table 1:** Mean (± SD) of the percentage of intratubular and intertubular hemorrhage (erythrocyte) in the testicular parenchyma of bulls submitted to 1, 3 and 6 testicular needle aspirations (TNA), every 30 days, using large (18G) or fine (22G) needles in the left or right testicle, respectively.

	Intratubular hemorrhage (%)		Intertubular hemorrhage (%)	
TNA	Left (18G)	Right (22G)	Left (18G)	Right (22G)
1 (G1)	4.1±5.8^Aa^	3.2±3.7^Aa^	21.6±20.3^Aa^	23.2±19.1^Aa^
3 (G2)	2.7±3.7^Aab^	2.5±2.6^Aa^	23.3±22.2^Aa^	18.0±20.0^Aa^
6 (G3)	0.0±0.0^Ab^	1.0±3.2^Aa^	8.8±11.6^Aa^	8.5±13.1^Aa^

18G: 40×12mm needle; 22G: 25×0.7mm needle.

A,B Different letters represent significant differences in the same line, according to each histological evaluation (P < 0.05).

a,b Different letters represent significant differences in the same column, according to the number of punctures within each histological evaluation (P < 0.05).

**Figure 3:**
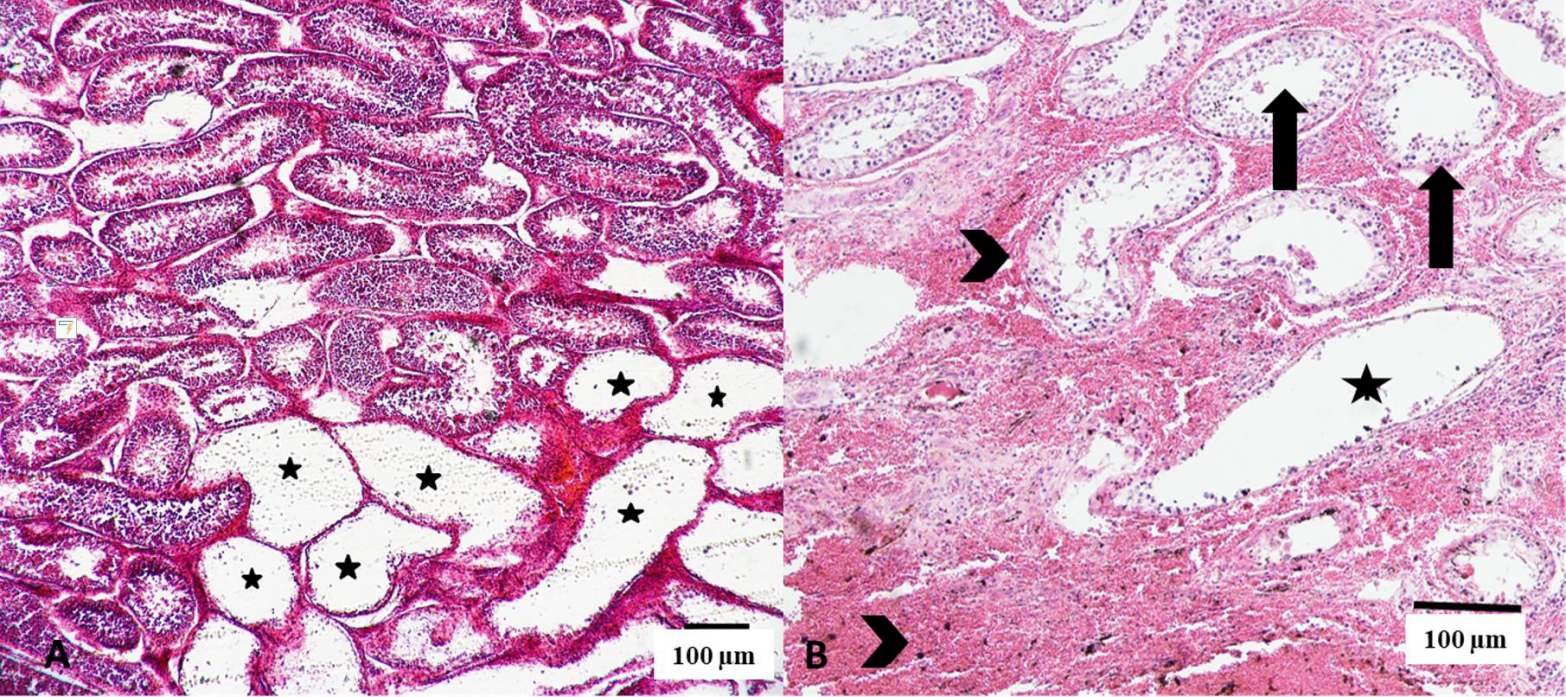
Photomicrograph of bovine testicular parenchyma after consecutive aspiration punctures illustrating areas of seminiferous tubule degeneration (A and B; stars), surrounded by normal and functional seminiferous tubules (B, arrows) and hemorrhagic areas (B, arrowheads). Hematoxylin and eosin stain.

No differences were observed in the percentage of degeneration of testicular parenchyma between G1, G2 and G3, regardless of the needle gauge used (P=0.7366). However, the use of different types of needles in TNA resulted in a higher proportion of degenerate seminiferous tubules using 40×12mm needles (26.03%) compared to 25×7mm needles (15.29%; P=0.0397). There was a significant interaction between the number of testicular punctures and the occurrence of seminiferous tubule degeneration when larger gauge was used (20.9%, 23% and 41.9%, respectively for G1, G2 and G3; Figure 3).

**Figure 4:**
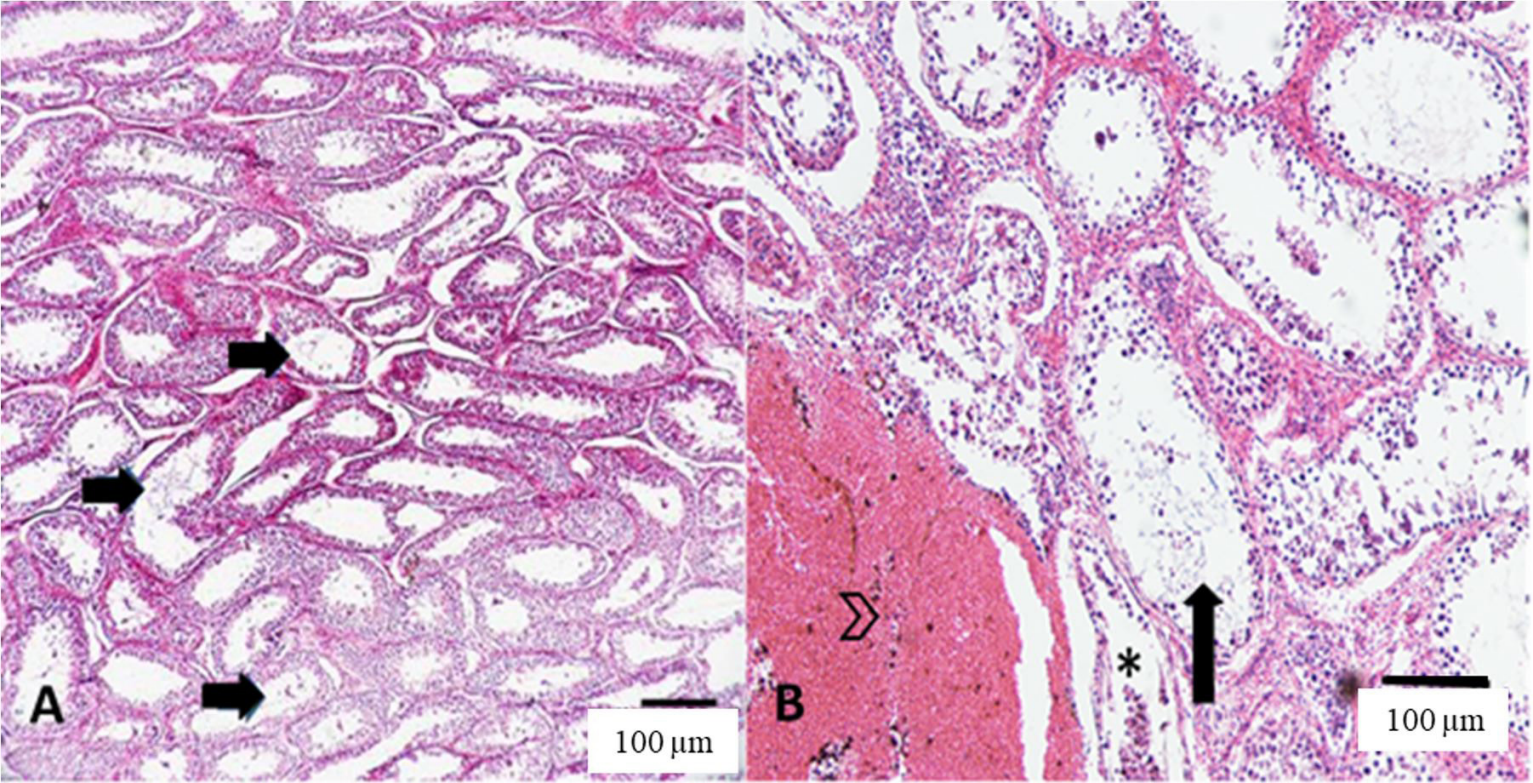
Photomicrographs of bovine testicular parenchyma after multiple testicular needle aspiration. (A) extensive areas of tubular dilation with intraluminal spermatozoa (arrows), indicating spermatogenic activity. (B) tubular degeneration (asterisk) and hemorrhagic areas around tubular dilatation (arrowheads). Hematoxylin and eosin stain.

When the needle size was not considered, a significant effect (P=0.0020) of the number of testicular punctures in the percentage of dilated seminiferous tubules was observed, with a reduction in the percentage of this lesion as a result of the increase in the number of punctures using fine needles (Table 2; Figure 4).

**Table 2:** Mean (± SD) of the percentage of tubular degeneration and dilation in the testicular parenchyma of bulls submitted to 1, 3 and 6 testicular needle aspirations (TNA), every 30 days, using large (18G) or fine (22G) needles in the left or right testicle, respectively.

	Tubular degeneration (%)		Tubular dilation (%)	
TNA	Left (18G)	Right (22G)	Left (18G)	Right (22G)
1 (G1)	20.9±17.4^Aa^	17.5±14.4^Aab^	13.4±8.9^Aa^	12.5±13.8^Aa^
3 (G2)	23.0±18.9^Aa^	9.5±12.1^Aa^	10.0±6.0^Aab^	10.0±10.0^Aa^
6 (G3)	41.9±30.2^Ab^	18.0±13.9^Bb^	6.9±4.6^Ab^	0.5±1.0^Bb^

18G: 40×12mm needle; 22G: 25×0.7mm needle.

A,B Different letters represent significant differences in the same line, according to each histological evaluation (P < 0.05).

a,b Different letters represent significant differences in the same column, according to the number of punctures within each histological evaluation (P < 0.05)

## Discussion

Cattle breeding success depends on the implementation of efficient reproductive programs capable of identifying different fertility problems that may compromise animal productivity (Chapwanya et al., 2008). Therefore, in addition to conventional andrological evaluations involving general clinical examination, reproductive examination and sperm analysis (Schrag and Larson, 2016), complementary techniques such as testicular needle aspiration biopsy (TNA) may provide more accurate information on spermatogenesis (Souza et al., 2004); and is indicated for fertility investigations involving oligozoospermia or azoospermia, also for the differential diagnosis of testicular pathologies (Papa and Leme, 2002). TNA allows the recovery of spermatogenic cells directly from the testis and can be used for several research purposes (Jha and Sayami, 2009; Mallidis and Baker, 1994), without depending on the collection and evaluation of semen as a semiological method for the evaluation of male fertility (Papa and Leme, 2002). The amount of spermatogenic cells obtained from TNA has a positive correlation with open testicular biopsies, demonstrating that testicular fine-needle aspiration is the method of choice for investigation of azoospermia in men (Adhikari, 2009; Jensen et al., 2016).

Although previous studies indicate that testicular TNA is not capable to cause deleterious effects on the testes, this technique is not often used for the diagnosis of animal infertility (Stelletta et al., 2011). Thus, one of the main concerns regarding testicular needle aspiration is the possibility of contamination by pathogenic microorganisms, which can cause inflammation, edema and induce degenerative processes (Gouletsou et al., 2012). However, no local or systemic clinical alterations that could indicate the occurrence of testicular pathological processes were identified in the present study, even in groups with repeated TNA. Similar results previously reported on canine (Gouletsou et al., 2010; Souza et al., 2004), feline (Gouletsou et al., 2012) and human species (Carpi et al., 2005; Raviv et al., 2004), indicate that this technique is minimally invasive, which can guarantee greater use of TNA as diagnostic method in animal andrology. According to Lewin et al. (1999) low rates of minor complaints were recorded after TNA (three cases of 85, 3.5%), demonstrating the safety of testicular aspiration for the recovery of spermatozoa in human patients with nonobstructive azoospermia.

Macroscopically, hemorrhagic areas in all orchiectomized testes were observed two days after the last TNA, independent of the experimental group. However, these areas may be considered as small, with no clinical or pathological relevance, as there was no bleeding or excessive fluid leakage to alter the scrotal circumference in G2 and G3 animals. Likewise, Gouletsou et al. (2012) verified that hemorrhage was a common finding in feline testes submitted to multiple punctures, and hemorrhagic processes appeared to occur in specific areas, indicating infiltration of erythrocytes in the interstitium, without the presence of large hematomas with the potential to alter the testicular dimensions.

Histologically, intra- and intertubular hemorrhage was also observed, the latter covering a larger area of the histological sections. These results are similar to those reported by James et al. (1979) and Gouletsou et al. (2010) and are justified by the trauma caused by the needles to the testicular microvasculature. Raviv et al. (2004) observed the formation of hematomas in about 6% of human patients submitted to TNA. However, all cases were self-limiting within 3 months after the procedure, indicating the transient and favorable clinical prognosis for patients with small haemorrhagic lesions after TNA.

In the present study, there was no effect of the needle gauge on the size of the intratesticular hemorrhagic process, demonstrating that even larger needles result in a discrete injury to local microvascularization. Likewise, Gouletsou et al. (2010) did not found effect of needle gauge (fine or large needle) on the occurrence and size of intratesticular hemorrhagic processes in canine species. However, there was a reduction in the size of the intratesticular hemorrhage in relation to the number of punctures performed in each testis. In addition to the erythrocyte uptake that may occur through the interstitium of the male gonad (Gouletsou et al., 2012), punctures were performed in the same testicular area. Thus, animals that were submitted to multiple TNA naturally had a greater extension of mechanical lesions and, consequently, localized areas of microfibrosis, with replacement of blood vessels by collagen. Therefore, the lower occurrence of intra- and intertubular hemorrhagic disorders of animals that received multiple TNA may be justified. This conclusion becomes more consistent from the analysis of tubular degeneration in the left testis of G3 animals. In this group, the occurrence of degenerative processes (above 40%) was the highest among the groups; however, intratubular hemorrhage was 0.0, suggesting the replacement of testicular parenchyma by local fibrosis.

Among all changes that occur in the testicular parenchyma during TNA, the alteration with the greater potential of injury is the degeneration of seminiferous tubules (Shufaro et al., 2002). James et al. (1979) reported the presence of hemorrhage, degeneration and necrosis in dog testes submitted to TNA without compromising spermatogenesis and testosterone synthesis. In our study, degenerative lesions in the testicular parenchyma restricted to areas along the needle path were also observed.

According to Figure 3, although areas of evident degeneration of the seminiferous tubules have been observed, the presence of spermatozoa in the tubular lumen indicates that the lesion did not extend along the testicular parenchyma. These findings confirm two important aspects related to the consequences of testicular punctures in cattle: 1) isolated degenerative changes by the passage of hypodermic needles; 2) the seminiferous epithelium surrounding this area maintains its normal spermatogenic capacity. Controversially, Shufaro et al. (2002) concluded that TNA induces severe, progressive and irreversible damage to the architecture of the seminiferous tubules, which eventually results in generalized and permanent tubular atrophy. However, these findings were established using rats as animal model for punctures; the small testicular volume of this species and the susceptibility to tissue and vascular lesions by the introduction of the needles during TNA may explain the different results.

No differences were observed in the percentage of degeneration of the testicular parenchyma when the three experimental groups were compared, regardless of the needle gauge used. These results allow concluding that TNA can be serially performed in cattle without compromising testicular health. In addition, the number of movements performed by the needles inside each testis during TNA may explain the low potential for injury to the testicular parenchyma. After insertion into the parenchyma, the needle was moved 3 times within each testis, as the methodology described by Gottschalk-Sabag et al. (1992), which concluded that with three movements per testis, the probability of causing degenerative lesions in the gonadal parenchyma would be lower. However, Gouletsou et al. (2012) dislocated the needle three or eight times within the testicular parenchyma and did not observe differences in the degree of testicular lesions in feline patients, suggesting that, even in the most invasive TNA, the incidence of gonadal lesions remains low.

In this study, the amount of degenerate seminiferous tubules was directly influenced by the needle size, with a higher proportion of lesions when a large needle was used. Although the use of large gauge needles may be recommended to obtain more spermatogenic cells (Carpi et al., 2006), our results indicate that thick needles should be avoided for multiple TNA in bulls.

In contradiction, Carpi et al. (2005) concluded that the use of large-gauge needles for TNA did not to produce relevant clinical or subclinical complications in human patients, presenting the same safety compared to the use of fine needles. However, the authors did not standardize the gauge of needles, using thicker needles (significantly thinner needles compared to those used in our study) for patients with greater testicular parenchyma volume. Probably this adaptation of the needle gauge to the testicular mass volume may minimize possible side effects related to punctures. In the same way, Gouletsou et al. (2010) did not observe differences in the occurrence and severity of testicular lesions in dogs submitted to testicular aspiration with fine or large needles. However, in this study, the caliber of the thick needles used was inferior to that used in our study, which may also explain the observed differences in the occurrence of lesions in the testicular parenchyma.

In conclusion, although lesions occur in the testicular parenchyma in bulls submitted to TNA, these alterations compromise the tissue in the path of the needle, without evidence of generalized lesions that could impair testicular health. Multiple testicular aspirations can be safely conducted with fine needles. The use of large needles should be encouraged for single or sporadic punctures, since frequent use may result in a higher percentage of tubular degeneration.
